# Brief oxygenation events in locally anoxic oceans during the Cambrian solves the animal breathing paradox

**DOI:** 10.1038/s41598-019-48123-2

**Published:** 2019-08-12

**Authors:** Tais W. Dahl, Marie-Louise Siggaard-Andersen, Niels H. Schovsbo, Daniel O. Persson, Søren Husted, Iben W. Hougård, Alexander J. Dickson, Kurt Kjær, Arne T. Nielsen

**Affiliations:** 1 GLOBE institute, University of Copenhagen, 1350 Copenhagen K, Denmark; 2 State Key Laboratories of Geological Processes and Mining Resources, China University of Geosciences, Wuhan, 430074 Wuhan, P.R. China; 3 Geological Survey of Denmark and Greenland (GEUS), 1350 Copenhagen K, Denmark; 4 Department of Plant and Environmental Sciences, University of Copenhagen, 1871 Frederiksberg C, Denmark; 5 Department of Earth Sciences, Royal Holloway University of London, Egham, Surrey TW20 0EX United Kingdom; 6 Department of Geosciences and Natural Resource Management, University of Copenhagen, 1350 Copenhagen K, Denmark

**Keywords:** Element cycles, Palaeontology, Element cycles

## Abstract

Oxygen is a prerequisite for all large and motile animals. It is a puzzling paradox that fossils of benthic animals are often found in black shales with geochemical evidence for deposition in marine environments with anoxic and sulfidic bottom waters. It is debated whether the geochemical proxies are unreliable, affected by diagenesis, or whether the fossils are transported from afar or perhaps were not benthic. Here, we improved the stratigraphic resolution of marine anoxia records 100–1000 fold using core-scanning X-Ray Fluorescence and established a centennial resolution record of oxygen availability at the seafloor in an epicontinental sea that existed ~501–494 million years ago. The study reveals that anoxic bottom-water conditions, often with toxic hydrogen sulfide present, were interrupted by brief oxygenation events of 600–3000 years duration, corresponding to 1–5 mm stratigraphic thickness. Fossil shells occur in some of these oxygenated intervals suggesting that animals invaded when conditions permitted an aerobic life style at the seafloor. Although the fauna evidently comprised opportunistic species adapted to low oxygen environments, these findings reconcile a long-standing debate between paleontologists and geochemists, and shows the potential of ultra-high resolution analyses for reconstructing redox conditions in past oceans.

## Introduction

Deposition of black shales was widespread in the Cambrian oceans (541–488 Ma)^1^. In the past decade, various geochemical redox proxies have been used to associate black shale facies to deposition under anoxic waters with hydrogen sulfide present in the water column. Basinal transects across continental shelves in various parts of the world outline a heterogeneous O_2_ landscape in the Cambrian ocean with oxic surface waters overlying anoxic mid-depth waters, akin to modern-day oxygen minimum zone settings, but sometimes with free hydrogen sulfide (H_2_S) present at mid-water depth and anoxic and ferruginous deep waters^2–4^. Areas with low O_2_ availability in the ocean are called ‘dead zones’ because all animals require O_2_ for respiration^5^. The expansion of dead zones was arguably an efficient kill mechanism during several of the most devastating animal crises in Earth history, including during the Cambrian^6,7^. When anoxia impinges on the seafloor, it changes the behavior of redox sensitive elements and leads to trace metal enrichments in the sediments. There is now evidence that the extent of seafloor anoxia waxed and waned over million year time scales^8–10^. Yet, the stability of marine redox conditions on shorter time scales has not been investigated in ancient shales due to low temporal resolution of previous paleoredox studies.

This problem has led to controversies between paleontologists and geochemists regarding strata where fossils of benthic animals are found in intervals containing geochemical evidence for anoxic bottom waters with poisonous H_2_S; e.g. the Alum Shale Formation of Scandinavia^8,10^. As a solution to this paradox, it has been suggested that *olenid* trilobites had chemoautotrophic symbionts and were specifically adapted to this habitat^11^, but this hypothesis has not been widely accepted. Here, we present evidence for a uniformitarian solution to the paradox, namely that anoxic sulfidic bottom waters were an intermittent rather than persistent feature, and that animals invaded the seafloor during oxygenated periods.

**Figure 1:**
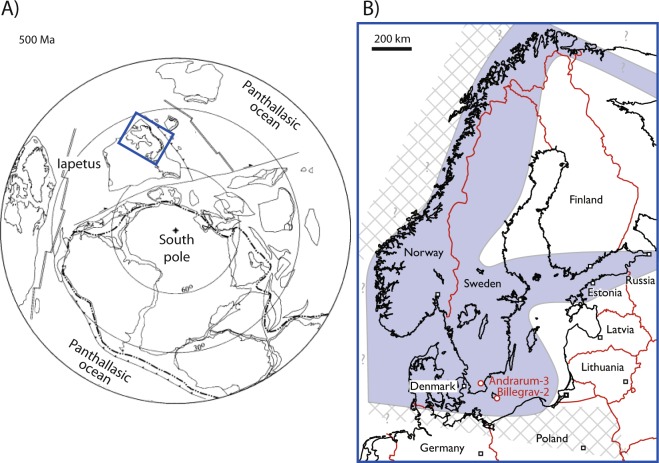
(**A**) Palaeogeographical reconstruction of the later Cambrian world^39^. (**B**) Geographical extent of the Alum Shale Sea (modified from ref.^29^), capitol cities (□), and location of wells referred to in this paper ().

**Figure 2:**
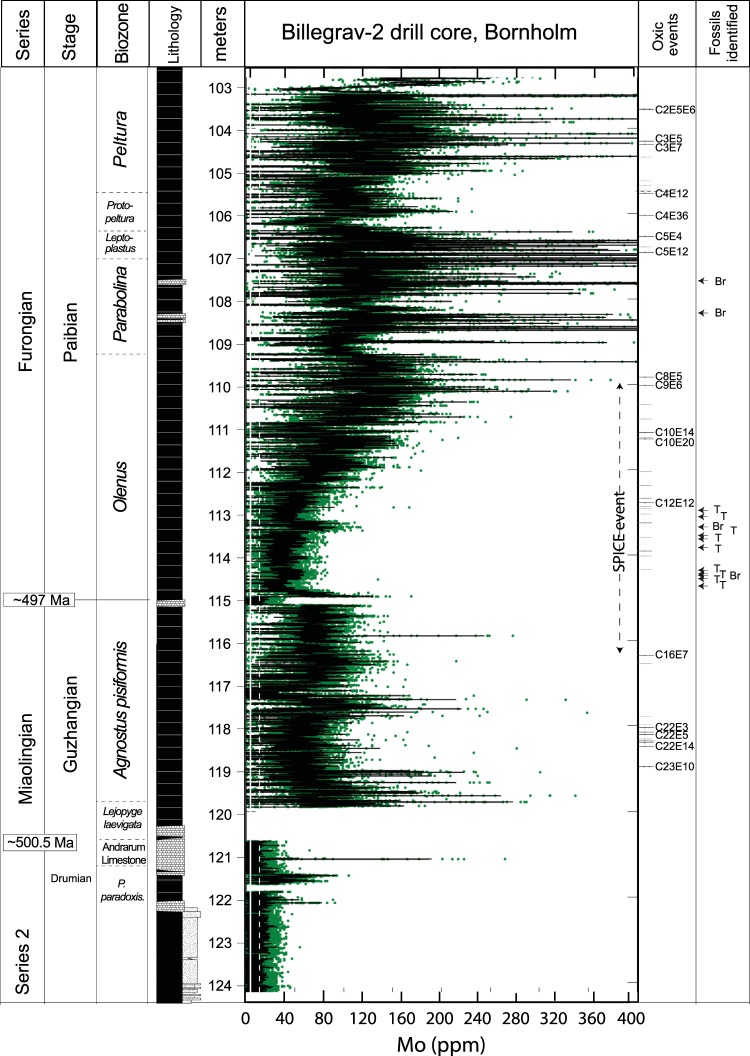
Stratigraphy of the Billegrav-2 drill core, Denmark, with Mo contents obtained using Core Scanning X-Ray Fluorescence spectrometry (0.2 mm resolution, 8 seconds per scan) on the outer surface of the core. The green dots represent Mo data obtained by XRF and the black curve represents the LOESS fit through the Mo data. A total of 51 oxygenation events are identified (grey, **–**) and the visually most prominent events are given a name (black, **—**). Fossil occurrences modified after ref.^30^. Br – brachiopod. T – Trilobite.

**Figure 3:**
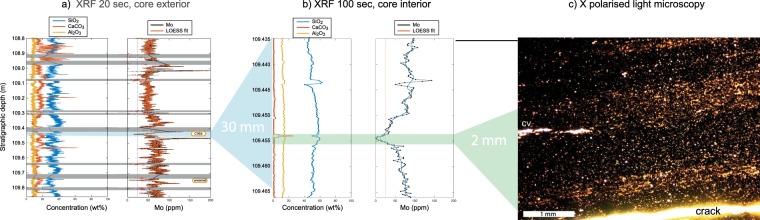
Example of oxygenation event (C8E5, 109.455 m), with Mo content obtained using XRF of (**a**) the core exterior (20 sec/scan), (**b**) the core interior (100 sec/scan) and (**c**) microscope photograph of the event as seen in thin section in cross-polarized light. Grey horizontal bars in (**a**) refer to intervals with no XRF data (e.g. cracks). CV = Calcite vein.

We used core scanning X-Ray Fluorescence spectroscopy (XRF) to record the sedimentary Mo contents of the Alum Shale to reconstruct bottom-water redox conditions in the late Cambrian Alum Shale Sea, where abundant benthic animal fossils have been found in strata with bulk-rock geochemical evidence for sulfidic-anoxic conditions at the seafloor^8,12^. Molybdenum is particularly useful in this respect because of its strong enrichment in sediments deposited from anoxic and sulfidic waters compared to oxic sediments and average crustal rock (~1 ppm)^12,13^. The Mo enrichment arises from the geochemical switch point at ~11 µM H_2_S, above which unreactive molybdate is converted to reactive thiomolybdates that are reductively removed with Fe-sulfides and/or organic matter from the water column (see Methods)^14–18^. The Mo profile from the late Cambrian Alum Shale in the Billegrav-2 drill core, Bornholm, Denmark (Fig. 1, see details in Methods section), follows a coherent curve with systematic trends preserved down to sub-millimeter resolution corresponding to a temporal resolution of only ~10^2^ years (Figs 2 and 3). Most of the core contains >100 ppm Mo that is characteristic of sediments deposited under anoxic and sulfidic water column conditions in the modern ocean, but low Mo contents in the Cambrian Series 2 interval are indicative of oxic bottom water conditions. Overall, the Mo curve shows the same meter-scale variability as recorded in the Andrarum-3 drill core from Scania, Sweden, including a famous Mo decline across the Miaolingian–Furongian boundary followed by an increase from the *Olenus* Superzone onwards^8^. This systematic decrease in Mo enrichment has previously been linked to a global marine Mo drawdown associated with a global expansion of anoxic water masses on the continental shelves, known as the ‘SPICE’ event^8,12^. Our new data from Billegrav-2 confirms that the muted Mo enrichments operated, at least, at a basin-wide scale in Scandinavia.

Remarkably, the new Mo curve also reveals rapid systematic fluctuations at (sub-) millimeter scale that have not previously been detected. Intervals with lower Mo contents in at least two consecutive measurement points (i.e. 0.4 mm thickness or more) relative to a baseline defined by adjacent layers with higher Mo contents were classified as ‘potential oxygenation events’ and those with a systematic Mo decline well below 25 ppm, characteristic of modern oxygenated seawater overlying reducing sedimentary porewaters^12,13^, and unbroken baselines are here counted as oxygenation events (e.g. Fig. 3a). A total of 449 potential oxygenation events were recorded in the 21.5 m of drill core, and 51 are regarded as oxygenation events. Most of these oxygenation events were only 1–5 mm thick (Fig. S5). Shorter events would escape recording due to our selection criteria and the resolution of the core scanner (0.2 mm).

**Figure 4:**
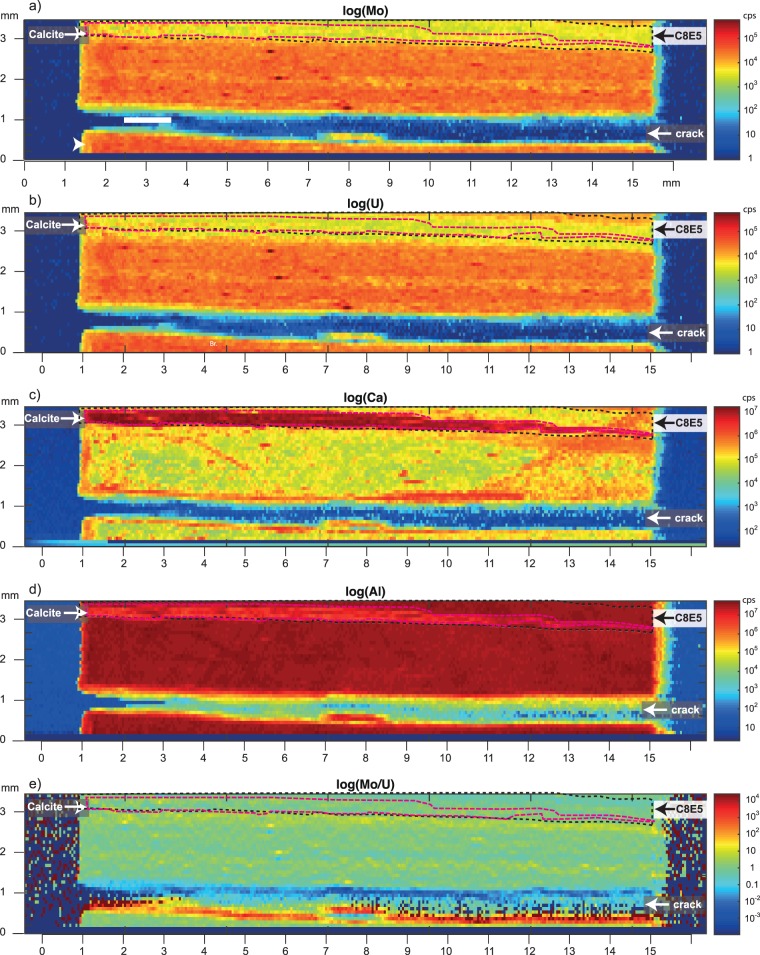
Laser Ablation ICP-MS data from event C8E5 showing (**a**) molybdenum (Mo), (**b**) uranium (U), (**c**) calcium (Ca), (**d**) aluminum (Al), and (**e**) Mo/U ratios. Colors in all panels are shown on a logarithmic scale. The regions with low Mo content and high Ca are indicated with black and pink dashed lines, respectively.

These brief oxygenation events were associated with neither changes in major element compositions nor with any visible sedimentological changes. Small Ca enrichments of presumably biogenic calcite were found in 14% of the events. Two-dimensional element mapping using LA-ICP-MS of oxygenation event ‘C8E5’ event at 109.85 m shows that calcium (located in carbonate minerals predominantly as CaCO_3_) is found exactly within the Mo minimum (Fig. 4). The origin of this material could not be uniquely determined, but it fills a crack that may result from dissolution of some sort of animal shell. The uranium (U) content concordantly followed the Mo drop as expected during seafloor oxygenation. Further, sedimentological changes or changes in the elemental compositions were relatively minor and dilution by carbonate played essentially no role on the dramatic changes in Mo and U contents. This is clearly demonstrated in C8E5 where the muted Mo and U enrichments persist throughout the bed and the adjacent calcitic infill (Fig. 4). The geochemical signals conclusively indicate that bottom water oxygenation took place and that this process opened up habitats for aerobic benthic fauna.

**Figure 5:**
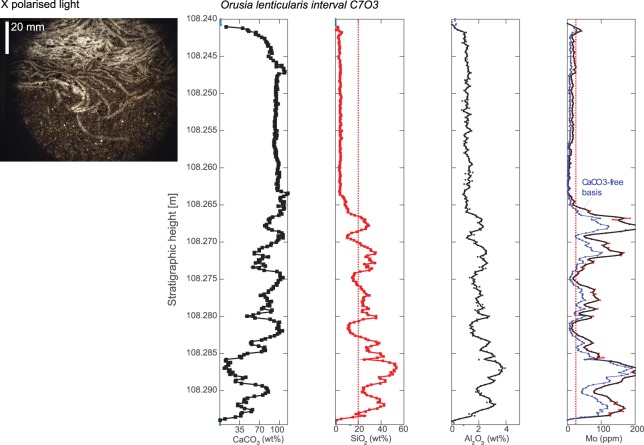
Stratigraphic Mo curve for the fossiliferous interval 108.30–108.24 m (‘C7O3’). Note: The upper part is fully oxygenated (low Mo) and rich in *Orusia* brachiopods (high Ca). The lower part displays strong inverse relationship between Ca and Mo contents. It should be kept in mind that the brachiopod shells are curved and cross the bedding plane, which mixes oxygenated and anoxic intervals.

To explore if oxygenation was always a driver for animal invasions, we investigated two fossiliferous intervals with abundant calcitic brachiopods (*Orusia lenticularis*) of 1 and 5 cm thickness (108.30–108.25 m, 107.515–107.505 m), respectively. The levels with the densest brachiopod populations were oxygenated, with low Mo concentrations (<10 ppm). However, both intervals also contain discrete levels with remarkably high Mo concentrations of 200–400 ppm that are indicative of average anoxic depositional conditions (Figs 5 and S6). Today, the highest Mo burial fluxes occur at the lowest H_2_S levels, where Mo-rich oxic waters meet anoxic and sulfidic bottom waters^19^. Therefore, intermittently euxinic settings with fluctuating O_2_/H_2_S levels produce the highest Mo enrichments, and brief oxygenation events in this interval would easily go undetected due to the curvature of the *O*. *lenticularis* shells and the rectangular analytical area of the core scanner (Figs 5 and S6). Importantly, there is a strong coupling between fossil content, as recorded by Ca content, and more oxygenated periods, as recorded by lower Mo contents (also on a CaCO_3_-free basis), which consistently points to oxygenation during times with the densest benthic animal populations.

## Driving Mechanism(s) for the Frequent Seafloor Oxygenation Events

The duration of the Mo events constrains the possible driving mechanism(s) for recurrent bottom water oxygenation. The average sedimentation rate (compacted values) for the ~500.5 to 497 Ma^20^ interval covering 120.7 to 115 m in the drill core is 1.6 mm/kyr. Therefore, a 2-mm-thick oxygenation event represents ~1200 yrs. A shorter duration is certainly possible due to non-uniformity of sediment accumulation rates. Both brachiopods (*Orusia lenticuaris*) and trilobites could spread quickly as they had planktonic (likely long-living) larvae as a possible adaption to cope with the harsh living conditions of the Paleozoic oceans. The fauna exhibits a specific preference for black shale environments, precluding a pelagic life style. It is also plausible that the animals living in the Alum Shale Sea produced vast amounts of pelagic larvae, enhancing their migration potential and likelihood of surviving somewhere in the basin^21^. We propose that whenever the conditions permitted it, the opportunistic Alum Shale fauna rapidly invaded the seafloor (e.g., within years or perhaps even months). Juveniles and adults are typically found together, suggesting that beneficial living conditions existed for enough time for one or more generations of animals to mature. Therefore, the oxygenated conditions probably prevailed for 1–1000 years, and unlikely for much longer than this. On this short time scale, the global marine Mo inventory would have been essentially constant, and we ascribe the oxygenation events to phenomena operating in the Alum basin^10^.

Bottom-water oxygenation can increase either as a result of overall higher O_2_ levels in the surface waters, slower aerobic consumption at depth, slower sulfate reduction at depth, and ventilation by O_2_-loaded waters brought into the deeper part of the basin. Oxygen levels in the surface ocean are governed by atmospheric pO_2_ and marine O_2_ solubility, as dictated by Henry’s law. The extremely short time scale of the oxygenation events precludes changes in atmospheric pO_2_ as the principle driver, because it varies too slowly over much longer time scales (>10^5^ y). Aerobic O_2_ consumption at depth varies as a function of photosynthetic productivity and organic export, which in turn is limited by the availability of the bio-limiting nutrients, e.g. phosphorus (P). Given that the basin was persistently marine and did not oscillate between restricted lagoonal and marine facies over oxygenation event time scales (e.g. linked to sea-level to changes), nutrients would have been constantly replenished by water exchange between the epicontinental sea and the open ocean. Thus, changes in marine P availability are also too slow (>10^4^ y) to explain the observed rapid changes. Brief reductions in the rate of organic export from the surface waters to the deeper part of the basin or a cessation of sulfide production via microbial sulfate reduction are difficult to reconcile with a known physical process. Instead, we infer that ocean stratification was periodically interrupted by deep-water ventilation in which oxygenated waters were pushed into the deeper part of the Alum Shale Sea, perhaps assisted by higher O_2_ solubility during colder periods. Climatic changes and changes in ocean-circulation patterns have not been established for the Cambrian hothouse climate^22^. Yet, we propose that these oxygenation events were climatically driven, for example linked to major storms or periods with high storm frequency. We envisage that lower atmospheric pO_2_ and concomitant dissolved O_2_ load in the surface waters of ~15–50% of today’s levels^10^ allowed for expansive anoxic waters impinging on the seafloor and that such conditions also made the seafloor more susceptible to oxygenation by changes in ocean ventilation and O_2_ solubility than in anoxic zones of the modern oceans.

In summary, our results offer a uniformitarian solution to a long-standing debate between paleontologists and geochemists for the oxygen availability and ecological requirements of animals living in organic-rich mudrock environments. The Olenid trilobites and a few brachiopods in the Alum Shale were adapted to live in low-O_2_ environments, but they still required O_2_ for breathing. The fauna was therefore adapted to opportunistically take advantage of the short-term re-oxygenation episodes embedded within a long-term de-oxygenated situation. Fossiliferous intervals are dominated by only 1–2 species that occur in immense numbers, whereas trilobite species known from better ventilated environments elsewhere are exceedingly rare in the Alum Shale^23–25^. In this way, frequent bottom-water ventilation in oxygen-restricted Cambrian oceans created intermittent habitats for opportunistic animals.

## Methods

### Samples and geological settings

The Alum Shale Formation is a marine succession deposited from the Miaolingian (‘middle’ Cambrian) through earliest Ordovician (Tremadocian). The unit originally covered some ~1,000,000 km^2^ of the Baltoscandian craton^26–28^. Deposition occurred after a pronounced rise in global sea level that led to flooding of the entire Scandinavian area and allowed slow deposition (average accumulation rates ~1–2 mm/ky, compacted values) of organic rich mud from the storm wave base and deeper in an extremely flat epicontinental sea. In the late Cambrian, Baltica was presumably located in the westerly wind belt at ~30–60°S paleolatitude that occasionally produced storm deposits in shoreface and innermost shelf areas^28,29^. During the Cambrian, notably the ‘early’ Cambrian (Terreneuvian–Cambrian series 2), and Early Ordovician, extensive formation of glauconite – a mixed Fe^2+^/Fe^3+^ mineral formed at the chemocline in modern oxygen minimum zones – took place in rather shallow water on the inner shelf ^28,29^.

We investigated a 21.7-m-thick interval (102.55–124.25 m) in the Billegrav-2 drill core from Bornholm, Denmark, that was deposited in an outer shelf setting more than ~500 km from the paleo-shoreline during the late Drumian to Paibian (‘middle’ to ‘late’ Cambrian) (Fig. 2)^30^. The drill core was retrieved in August 2010 by the Geological Survey of Denmark and Greenland and has since then been stored under dry conditions in the core repository^31^.

The stratigraphic succession consists of typical laminated Alum Shale interbedded by a few thin limestone horizons (early diagenetic) (Fig. 2). The shales on Bornholm are similar to the Alum Shale deposited elsewhere in Scandinavia at this time with geochemical characteristics similar to that of modern sediments deposited under anoxic and sulfidic bottom waters. Prominent features include high total organic carbon (average ~10 wt%), 100-fold enrichments in redox sensitive trace metals (including >100 ppm Mo and up to 300 ppm U) relative to average crustal abundance (1.5 and 2.8 ppm, respectively)^8,12,32–34^ and a high proportion of highly reactive iron (Fe_HR_/Fe_T_ > 0.9) of which most is pyrite (Fe_P_/Fe_HR_ > 0.9)^8,10^. Nevertheless, the shales also host an abundant, although low-diversity, fauna of olenid and agnostoid arthropods and brachiopods, which are only known from similar black shale deposits. This benthic fauna is generally interpreted to have been adapted to low O_2_ levels, although some authors have proposed that they lived in anoxic waters in the presence of hydrogen sulfide – toxic to most animals today^11^. It has also been proposed that the fossils might be allochthonous^35^, but the general lack of size sorting precludes this interpretation.

A systematic decline from ~100 ppm Mo to ~50 ppm across the Miaolingian–Furongian boundary (~497 Ma) has been linked to a global drawdown of the oceanic Mo inventory during the ‘SPICE’ event, which presumably represents an expansion of anoxic water masses at global scale^8,9^. Still, there have been no previous attempts to geochemically explore the persistence of anoxia in any of the many anoxic basins around the world.

### Analytical methods

Non-destructive and continuous analyses at sub-millimeter stratigraphic resolution were performed at the Center for Geogenetics at University of Copenhagen using an Itrax XRF core scanner from Cox Analytical Systems^36^ equipped with a rhodium (Rh) tube as the X-Ray source. The measurements were acquired at a vertical resolution of 0.2 mm and horizontal width of 10 mm. The analyzed area per scan was 2 mm^2^ and 10–100 fold smaller than previous Mo XRF analyses (12–100 mm^2^)^12,37^. The strata in the core are tilted no more than 10°, leading to a stratigraphic resolution better than 0.23 mm. The analyses were first performed on the outer, round surface of the core (55 mm core diameter) in 24 segments of ~1 m length giving a total of 97,876 analyses including gaps between core pieces and 73,863 lines excluding gaps. Gaps were recognized as lines with Si <10 wt% in the shale. The exposure time was mostly 8 seconds per scan (7–20 sec/scan). The applied current and voltage was 30 kV and 50 mA, respectively. Hereafter, the core was cut through the center at selected intervals and data were collected for even longer exposure times (100 s per scan) across 24 intervals covering pieces 4–10 cm long where the Mo signal systematically decreased and/or benthic animal fossils were found.

The absorption peaks were identified and peak areas were quantified using the Q-Spec software from Cox Analytical Systems. Molybdenum was determined at the Mo Ka absorption edge at 17.5 keV (Fig. S1), where there are no atomic interferences. The photon count for XRF analysis is, however, known to vary as a function of sediment inhomogeneity and varying physical properties. To quantify these effects and provide quantitative Mo abundances, we performed a series of control experiments: (1) Reference rock powders with variable matrix (basalts, Alum Shale, and other sediments) were analyzed and (2) a gravimetric standard curve was constructed based on samples with the same fine-grained sediment matrix mixed with precisely weighed amounts of molybdenite, MoS_2_. This six-point gravimetric standard curve with constant matrix composition demonstrates a perfect linear relationship (R^2^ = 0.999, p = 10^−7^) between known Mo concentrations from 0 to 200 ppm and observed peak area normalized to Rh Coh (Fig. S1). Powdered rock materials with variable physical and chemical matrix properties yield a reduced signal by <32% compared to the gravimetric curve (Fig. S2, Supplementary Dataset). Therefore, we infer that changes in sediment matrix can also affect the Mo signal and produce misleading results.

To ensure that the XRF Mo signal from the drill core samples reflects true Mo variability and that accurate concentrations can be reported, we determined Mo contents for a subset of drill core samples using two independent methods.

First, we cut ultra-thin (0.4–0.5 mm) slices of drill core samples along the bedding plane using a 0.25-mm-thick diamond string across two events where Mo contents dropped dramatically from ~300 ppm to ~20 ppm (C2E5E6 and C9E6). High precision molybdenum concentration measurements were performed on powdered core cuts and saw dust by adding a precise mass of ^100^Mo-^97^Mo double spike to samples containing 50–200 ng natural Mo, digested and purified using ion chromatography according to the methods described in ref.^38^ before being analyzed using a Neptune Multi-Collector Inductively Coupled Mass Spectrometry (MC-ICP-MS) at Royal Holloway University of London. Concentrations are calculated by isotope dilution from the ^100^Mo/^95^Mo ratio. A good correlation was found between Mo contents obtained in Q-SPEC using a calibration standard (SGR-1) and the isotope dilution data, Mo_ICPMS_ = 2 · Mo_XRF_ −5 (ppm) (Figs S3 and S4). The slope deviates substantially from unity and demonstrates the need for independent control measurements of the analyzed material in order to establish accurate Mo profiles from core-scanning XRF. All Mo data reported here is corrected according to this calibration.

Secondly, we used Laser Ablation ICPMS at the Department of Plant and Environmental Sciences, University of Copenhagen, and mapped the concentration of Mo, U, Ca and Al across another event, where the Mo content dropped from ~80 to ~0 ppm (C8E5, Fig. 3). The ablated area, covering a total of 14 × 42 mm, was analyzed continuously on a point by point basis at a set speed of 200 µm/s with each data point covering an area of 80 × 50 µm (stratigraphic height × width). Elemental maps are shown in Fig. 4.

## Supplementary information

Supplementary figures

Supplementary dataset

## Data Availability

All data needed to evaluate the conclusions in the paper are present in the paper and/or Supplementary Materials. Additional data and models related to the paper may be requested from the corresponding author.

## References

[1] Berry W, Wilde P (1978). Progressive ventilation of the oceans – an explanation for the distribution of the lower Paleozoic black shales. American Journal of Science.

[2] Jin C (2016). A highly redox-heterogeneous ocean in South China during the early Cambrian (∼529–514 Ma): Implications for biota-environment co-evolution. Earth and Planetary Science Letters.

[3] Hammarlund EU (2017). Early Cambrian oxygen minimum zone-like conditions at Chengjiang. Earth and Planetary Science Letters.

[4] Guilbaud, R. *et al*. Oxygen minimum zones in the early Cambrian ocean. *Geochemical Perspectives Letters*, 33–38, 10.7185/geochemlet.1806 (2018).

[5] Diaz RJ, Rosenberg R (2008). Spreading dead zones and consequences for marine ecosystems. Science.

[6] Zhang F (2018). Congruent Permian-Triassic δ238U records at Panthalassic and Tethyan sites: Confirmation of global-oceanic anoxia and validation of the U-isotope paleoredox proxy. Geology.

[7] Jost AB (2017). Uranium isotope evidence for an expansion of marine anoxia during the end-Triassic extinction. Geochemistry, Geophysics, Geosystems.

[8] Gill BC (2011). Geochemical evidence for widespread euxinia in the later Cambrian ocean. Nature.

[9] Dahl TW (2014). Uranium isotopes distinguish two geochemically distinct stages during the later Cambrian SPICE event. Earth and Planetary Science Letters.

[10] Dahl TW (2010). Devonian rise in atmospheric oxygen correlated to the radiations of terrestrial plants and large predatory fish. Proceedings of the National Academy of Sciences of the United States of America.

[11] Fortey R (2000). Olenid Trilobites: The Oldest Known Chemoautotrophic Symbionts?. Proceedings of the Natural Academy of Sciences.

[12] Dahl TW (2013). Tracing euxinia by molybdenum concentrations in sediments using handheld X-ray fluorescence spectroscopy (HHXRF). Chemical Geology.

[13] Scott C, Lyons TW (2012). Contrasting molybdenum cycling and isotopic properties in euxinic versus non-euxinic sediments and sedimentary rocks: Refining the paleoproxies. Chemical Geology.

[14] Ericksson B, Helz G (2000). Molybdenum(VI) speciation in sulfidic waters: Stability and lability of thiomolybdates. Geochimica et Cosmochimica Acta.

[15] Dahl TW, Chappaz A, Fitts JP, Lyons TW (2013). Molybdenum reduction in a sulfidic lake: Evidence from X-ray absorption fine-structure spectroscopy and implications for the Mo paleoproxy. Geochimica et Cosmochimica Acta.

[16] Dahl TW (2017). Evidence of molybdenum association with particulate organic matter under sulfidic conditions. Geobiology.

[17] Helz GR, Bura-Nakić E, Mikac N, Ciglenečki I (2011). New model for molybdenum behavior in euxinic waters. Chemical Geology.

[18] Scholz F, Siebert C, Dale AW, Frank M (2017). Intense molybdenum accumulation in sediments underneath a nitrogenous water column and implications for the reconstruction of paleo-redox conditions based on molybdenum isotopes. Geochimica et Cosmochimica Acta.

[19] Algeo TJ, Lyons TW (2006). Mo-total organic carbon covariation in modern anoxic marine environments: Implications for analysis of paleoredox and paleohydrographic conditions. Paleoceanography.

[20] Ogg, J. G., Ogg, G. & Gradstein, F. M. *A concise geologic time scale: 2016*. (Elsevier, 2016).

[21] Nielsen AT (1997). A review of Ordovician agnostid genera (Trilobita). Earth and Environmental Science Transactions of The Royal Society of Edinburgh.

[22] Elrick M, Rieboldt S, Saltzman M, McKay RM (2011). Oxygen-isotope trends and seawater temperature changes across the Late Cambrian Steptoean positive carbon-isotope excursion (SPICE event). Geology.

[23] Żylińska A, Weidner T, Ahlgren J, Ahlberg P (2015). Exotic trilobites from the uppermost Cambrian Series 3 and lower Furongian of Sweden. Acta Geologica Polonica.

[24] Henningsmoen, G. J. S. U. A. D. N. V.-A. I. O., I, Matematisk-naturvidenskapelig Klasse. The trilobite family Olenidae: With description of Norwegian material and remarks on the Olenid and Tremadocian Series. 1–303 (1957).

[25] Schovsbo N (2001). Why barren intervals? A taphonomic case study of the Scandinavian Alum Shale and its faunas. Lethaia.

[26] Cocks LRM, Torsvik TH (2005). Baltica from the late Precambrian to mid-Palaeozoic times: The gain and loss of a terrane’s identity. Earth-Science Reviews.

[27] Blakey, R. C. In *Special Paper 441: Resolving the Late Paleozoic Ice Age in Time and Space* 1–28 (2008).

[28] Nielsen AT, Schovsbo NH (2015). The regressive Early-Mid Cambrian ‘Hawke Bay Event’ in Baltoscandia: Epeirogenic uplift in concert with eustasy. Earth-Science Reviews.

[29] Nielsen AT, Schovsbo NH (2011). The Lower Cambrian of Scandinavia: Depositional environment, sequence stratigraphy and palaeogeography. Earth-Science Reviews.

[30] Nielsen A, Schovsbo N, Klitten K, Woollhead D, Rasmussen C (2018). Gamma-ray log correlation and stratigraphic architecture of the Cambro-Ordovician Alum Shale Formation on Bornholm, Denmark: Evidence for differential syndepositional isostasy. Bulletin of the Geological Society of Denmark.

[31] Schovsbo N, Nielsen A, Klitten K, Mathiesen A, Rasmussen P (2011). Shale gas investigations in Denmark: Lower Palaeozoic shales on Bornholm. Geological Survey of Denmark and Greenland Bulletin.

[32] Schovsbo NH (2002). Uranium enrichment shorewards in black shales: A case study from the Scandinavian Alum Shale. GFF.

[33] Taylor S, McLennan S (1995). The geochemical evolution of the continental crust. Reviews of Geophysics.

[34] Buchardt B, Clausen J, Thomsen E (1986). Carbon isotope composition of Lower Palaeozoic kerogen: Effects of maturation. Advances in Organic Geochemistry.

[35] Dworatzek, M. *Sedimentology and petrology of carbonate intercalations in the Upper Cambrian Olenid shale facies of southern Sweden*. Vol. 81 (Sveriges geologiska undersökning, 1987).

[36] Croudace, I., Rindby, A. & Rothwell, R. ITRAX: description and evaluation of a new multi-function X-ray core scanner. *Geological Society, London, Special Publications* New Techniques in Sediment Core Analysis 51–63 (2006).

[37] Wirth SB (2013). Combining sedimentological, trace metal (Mn, Mo) and molecular evidence for reconstructing past water-column redox conditions: The example of meromictic Lake Cadagno (Swiss Alps). Geochimica et Cosmochimica Acta.

[38] Dickson A, Jenkyns H, Porcelli D, Boorn SVD, Idiz E (2016). Basin-scale controls on the molybdenum-isotope composition of seawater during Oceanic Anoxic Event 2 (Late Cretaceous). Geochimica et Cosmochimica Acta.

[39] Cocks L, Torsvik T (2002). Earth geography from 500 to 400 million years ago: a faunal and palaeomagnetic review. Journal of the Geological Society, London.

