# Supplementary material for: Brief oxygenation events in locally anoxic oceans during the Cambrian solves the animal breathing paradox
Source: Sci Rep. 2019 Aug 12;9:11669. doi: 10.1038/s41598-019-48123-2 (PMC6690889; doi:10.1038/s41598-019-48123-2)
Supplement: Supplementary file 1 — Supplementary figures [file 41598_2019_48123_MOESM1_ESM.pdf]

SUPPLEMENTARY INFORMATION

Supplementary figures to:

Brief oxygenation events in locally anoxic oceans during the Cambrian solves the animal breathing paradox

Tais W. Dahl[1,2*], Marie-Louise Siggaard-Andersen[3], Niels H. Schovsbo[4], Daniel O. Persson[5], Søren Husted[5], Iben W. Hougård[1], Alexander J. Dickson[6], Kurt Kjær[3], Arne T. Nielsen[1].

1) GLOBE institute, University of Copenhagen, 1350 Copenhagen K, Denmark

2) State Key Laboratories of Geological Processes and Mining Resources, China University of Geosciences, Wuhan, 430074 Wuhan, P.R. China

3) Biological Institute, Center for GeoGenetics, University of Copenhagen, 1350 Copenhagen K, Denmark

4) Geological Survey of Denmark and Greenland (GEUS), 1350 Copenhagen K, Denmark

5) Department of Plant and Environmental Sciences, University of Copenhagen, 1871 Frederiksberg C, Denmark

6) Department of Earth Sciences, Royal Holloway University of London, Egham, Surrey, TW20 0EX, United Kingdom

*Corresponding author: tais.dahl@ign.ku.dk, ORCID ID: 0000-0003-4629-8036

1. Supplementary figures

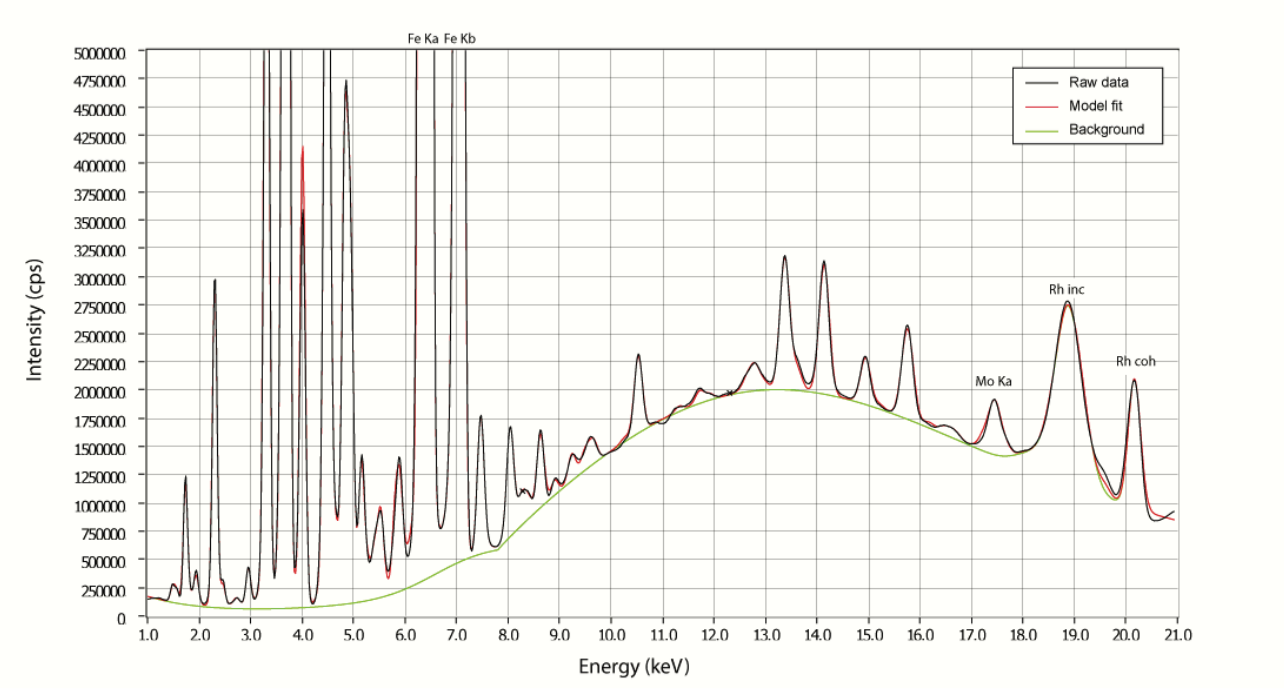

Figure S1. Characteristic XRF spectrum obtained with Itrax Core Scanning XRF. Molybdenum is detected at the Mo Kα absorption peak (17.4 keV), which is free of atomic interferences. Rh coh = rhodium coherent peak (elastic scattering), Rh inc = rhodium incoherent peak (inelastic scattering).

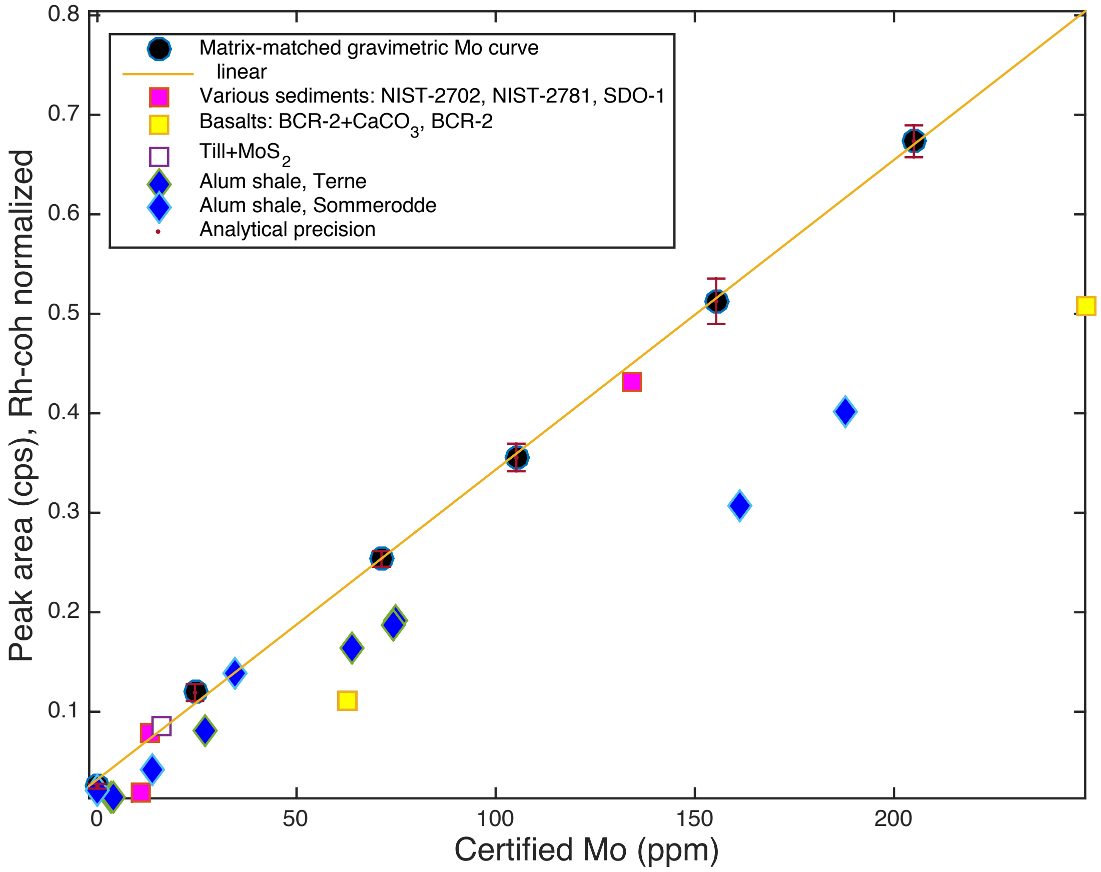

Figure S2. Calibration curves for Mo content in various reference materials. See legend and Methods for details.

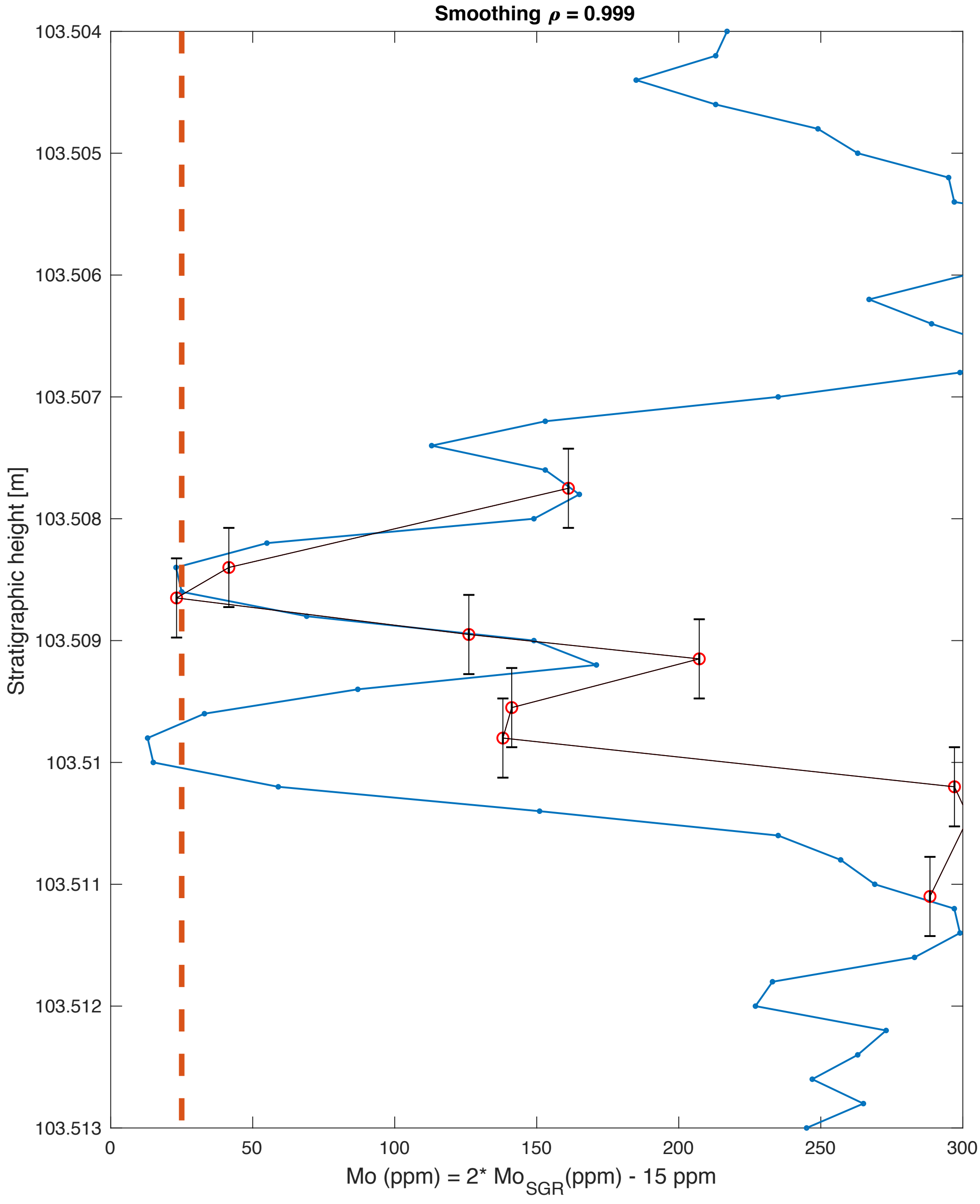

Figure S3. Molybdenum contents measured by XRF (core interior, 100 sec/scan, blue curve) and high precision ID-ICP-MS data obtained at Royal Holloway University of London[40] (red circles) for the two consecutive oxygenation events called 'C2E5E6.

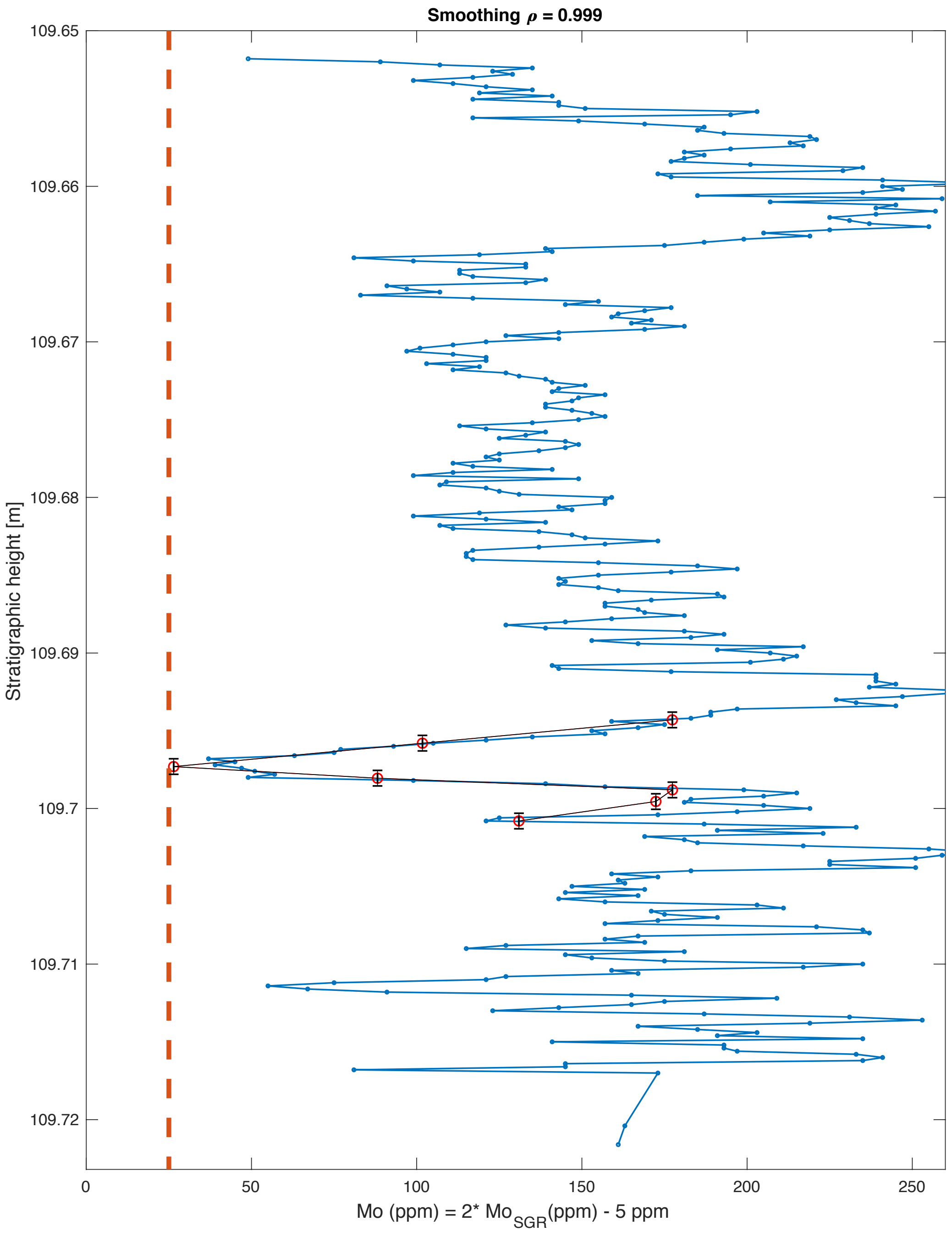

Figure S4. Molybdenum contents measured by XRF (core interior, 100 sec/scan, blue curve) and high precision ID-ICP-MS data obtained at Royal Holloway University of London[40] (red circles) for oxygenation event 'C9E6'.

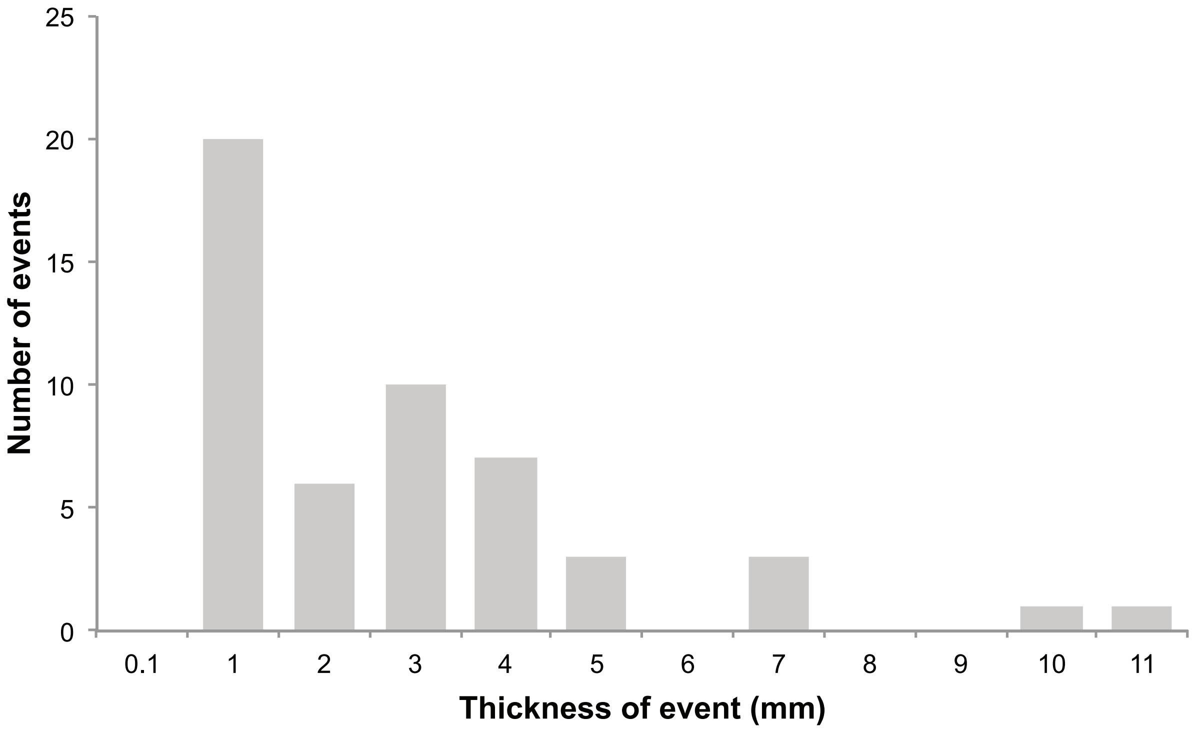

Figure S5. Histogram of oxygenation event thickness for the 51 oxygenation events in the studied interval of the Billegrav-2 drill core.

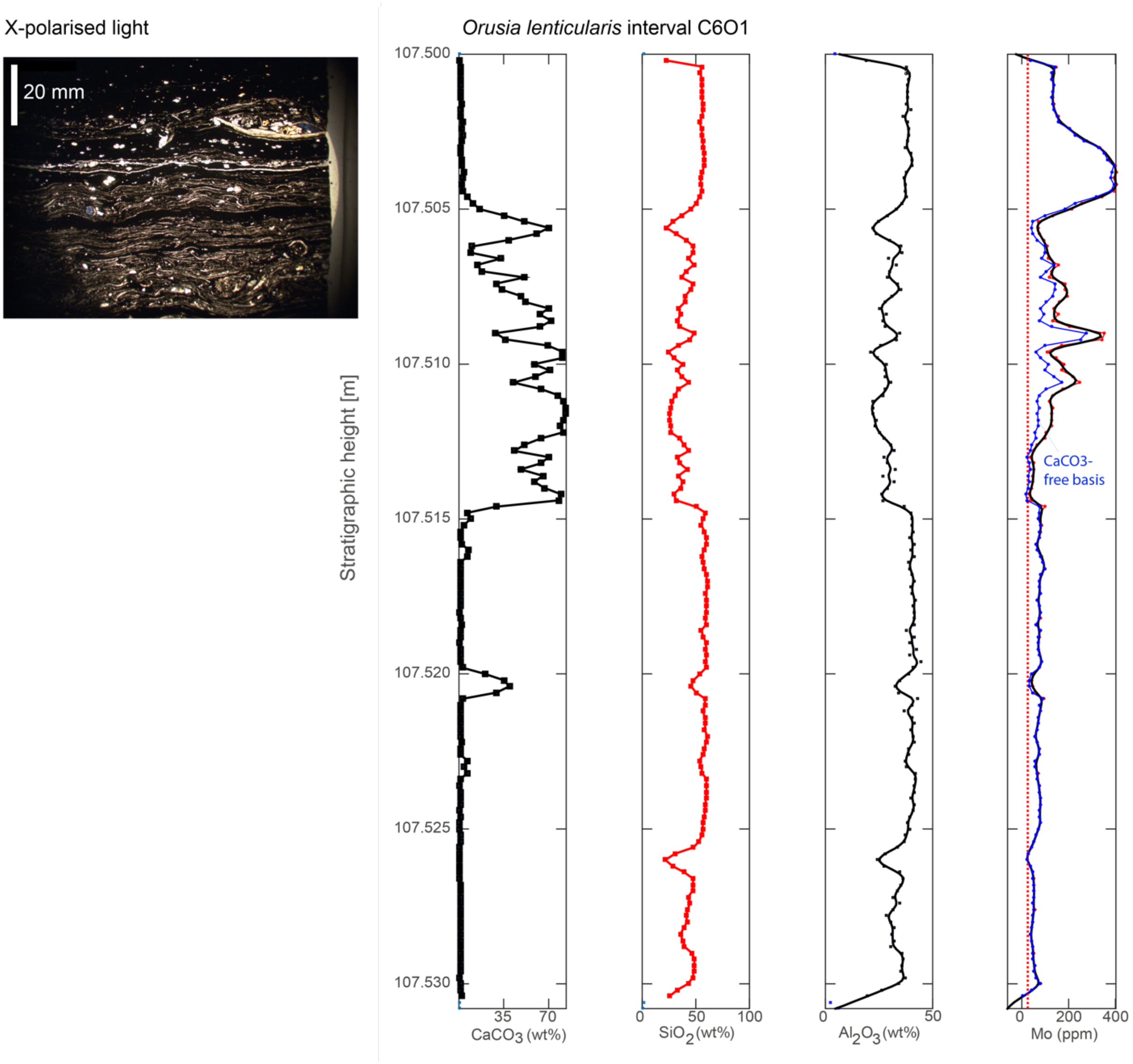

Figure S6. Stratigraphic Mo curve for the fossiliferous interval 107.50–107.53 m ('C6O1').
